# Observed birth prevalence of congenital anomalies among live births at a regional facility in KwaZulu Natal Province, South Africa

**DOI:** 10.1371/journal.pone.0255456

**Published:** 2021-08-03

**Authors:** Muhammad Zubayr Saib, Barnesh Lalloo Dhada, Colleen Aldous, Helen Louise Malherbe

**Affiliations:** 1 KwaZulu Natal Department of Health, Paediatrics and Child Health, Grey’s Hospital, Pietermaritzburg, South Africa; 2 Paediatrics & Child Health, Nelson R Mandela School of Clinical Medicine, College of Health Sciences, University of KwaZulu Natal, Durban, South Africa; 3 School of Clinical Medicine, College of Health Sciences, University of KwaZulu Natal, Durban, South Africa; 4 KwaZulu Natal Research Innovation and Sequencing Platform (KRISP), School of Laboratory Medicine and Medical Sciences, College of Health Sciences, University of KwaZulu Natal, Durban, South Africa; 5 Rare Diseases South Africa NPC, Johannesburg, South Africa; Ohio State University, UNITED STATES

## Abstract

Congenital disorders (CDs), defined as abnormalities in structure or function present at birth, are an important contributor to the disease burden in developing countries. The size and extent of the problem in South Africa (SA) are unknown due to the lack of recent, reliable, observed data on CDs. To address this empirical data gap, this study aimed to measure the birth prevalence of congenital anomalies (a sub-set of CDs) and to describe the pattern of these anomalies at a regional hospital in KwaZulu Natal (KZN), SA. A retrospective, observational, descriptive review of congenital anomalies diagnosed within the neonatal service at Edendale Hospital (EDH), KZN was undertaken between January and December 2018. All EDH in-house live births diagnosed and notified with congenital anomalies by discharge were included. Stillbirths, other pregnancy losses and out-born neonates were excluded. Data were actively collected from the birth register, neonatal admission register, and the individual paper-based surveillance tool developed by the National Department of Health. The in-facility birth prevalence rate for congenital anomalies was 15.57 per 1 000 live births. The most observed system was musculoskeletal (32%) followed by circulatory system anomalies (19%). When the observed birth prevalence rates of key congenital anomalies were compared with previously published, modelled South African data, no significant difference was found. This study responds to the paucity of birth prevalence data on CDs overall and offers evidence that obvious, structural CDs (congenital anomalies) need to be addressed in the SA public health system.

## Introduction

Congenital disorders (CDs) are defined as structural or functional abnormalities of prenatal origin which are present at birth [1]. While the majority of CDs are due to genetic or partially genetic causes occurring pre-conception, a proportion occurs after conception due to abnormalities of the foetal environment, while the cause of many remains unknown [2]. As a group of conditions, CDs are a major contributor to the global burden of disease, with an estimated 5 million births affected by serious CDs, which result in death or lifelong disability in the absence of care. Global estimates for 2010–2014 indicated over 400 000 foetal deaths, 2.5 million under-five deaths and a further 2 million children surviving at 5 years of age with significant disability attributed to CDs [3]. In 2010, the World Health Assembly (WHA) reaffirmed the importance of CDs as a healthcare issue through the adoption of Resolution WHA 63.17 and outlined actions for their management and prevention [4]. These remain relevant to achieving Goal 3 targets of the Sustainable Development Goals (SDGs) to decrease neonatal and infant mortality rates and preventable under-five deaths by 2030 [5]. Many of these actions are yet to be implemented by member states, including South Africa (SA).

While CDs affect all populations worldwide, the scale of the burden varies between populations. True differences in these rates may be due to varying maternal age distribution for chromosomal disorders, consanguinity practices affecting recessive, single-gene disorders and localised environmental factors (teratogens) [6]. The birth prevalence of *most* congenital anomalies—a distinct sub-set of CDs including obvious, structural malformations only [1], remains similar between populations. Notable exceptions do occur, such as isolated neural tube defects for which lower birth prevalence has been observed in Sub-Saharan Africa [6–8]. The greatest mortality and morbidity resulting from CDs is seen in low and middle-income countries (LMIC), with apparent differences in CD birth prevalence rates between these resource-limited countries attributed to varied diagnostic, care and prevention services and underreporting to varying degrees [9].

Quantifying the CD burden of disease has been underway for decades in many high-income countries (HIC) using empiric datasets collected through established surveillance systems, such as the European Registration of Congenital Anomalies (EUROCAT) [10]. Analyses of these data enable healthcare policymakers to develop and implement appropriate medical genetic services in response, for the care and prevention of those affected by CDs. However, in LMIC, empiric CD data are inadequate, unreliable or missing. While modelled data serve as a valuable tool in the interim, the long-term collection of empiric CD data is necessary. This requires relevant investment and training to enable accurate, timely diagnoses and the development of reliable surveillance systems [11]. In SA, a recent, comprehensive evaluation of the full burden of disease represented by CDs is lacking. Concerted actions were undertaken in the late 1990s and early 2000s to develop medical genetic services, including surveillance, as CDs began to emerge as an important cause of child mortality and morbidity. This commitment waned with the rise of the Human Immunodeficiency Virus/Acquired Immunodeficiency Syndrome (HIV/AIDS) epidemic as a competing health priority [12–14]. Data published in 2016 from the current birth defect surveillance system implemented by the SA National Department of Health (NDoH) since 2006 highlighted inconsistent and unreliable data with significant underreporting of CDs compared to modelled estimates [15]. With the successful management of HIV/AIDS, particularly the prevention of mother to child transmission (PMTCT) and immunisation for other infectious diseases, SA is undergoing a positive epidemiological transition once again as CDs re-emerge as a key cause of neonatal, infant and child deaths [12,16–18].

To fill the gap in empiric CD data in the country, and confirm the estimated CD disease burden this study aimed to: 1) Measure the birth prevalence of congenital anomalies among live births—a sub-set of CDs as categorised in Chapter XVII: Congenital Malformations, Deformations, and Chromosomal Abnormalities in the International Classification of Diseases and Related Health Problems (ICD-10) [19]; and 2) To describe the pattern of congenital anomalies observed, at a regional hospital in KwaZulu Natal (KZN) Province in 2018 using the Birth Defects Notification Tool (BDNT) developed and administered by the NDoH. Collected data and birth prevalence rates were compared with existing published data and modelled estimates for key congenital anomalies in SA. The study also included a quality improvement component to promote and maintain accurate data as part of routine clinical care.

## Method

### Design

This study was a retrospective, observational, descriptive review of congenital anomalies diagnosed at birth within the neonatal service at Edendale Hospital (EDH) in KZN Province, SA. The study period was from 1st January 2018 to 31st December 2018.

### Study setting

EDH is a regional (secondary level) healthcare facility located in the uMgungundlovu District in the city of Pietermaritzburg. It serves a predominantly urban population of around 1.4 million mainly indigenous, Zulu-speaking African people. It has well-organized obstetric services and a 62-bed Neonatal Care Unit (NCU) catering for an average of 600 in-facility deliveries a month and referrals from the surrounding state-run healthcare clinics. This includes normal and complicated deliveries, with newborn care for well-babies and those requiring intensive care. This arrangement of a single, referral facility providing healthcare services to a relatively unchanging catchment population provided a relevant setting to meet the objectives of this study.

### Study participants

As part of routine care, all in-house live births at EDH underwent a comprehensive neonatal examination within 24 hours of birth. Those identified with congenital anomalies were offered appropriate care and the BDNT was completed. These clinical records were included in the scope of this study, regardless of the gestational age of the baby. Stillbirths and other pregnancy losses (spontaneous/induced abortions, including termination of pregnancy due to foetal abnormality) were excluded as little routine clinical data were collected for these cases. Neonates with identified congenital anomalies born elsewhere and referred to EDH after birth were also excluded from the study to avoid inflating the birth prevalence rate as the number of deliveries at referral sites was not accessible within the study.

### Case definition

For this study, *congenital anomalies* only (a sub-set of CDs) were recorded. Congenital anomalies are defined as structural or anatomical abnormalities detected at birth (by discharge in this study) and classified according to Chapter XVII of ICD-10 [19]. Based on this case definition, functional anomalies and other CDs listed elsewhere in the ICD-10 system that are not easily recognisable (i.e., without obvious dysmorphology) at birth remained unidentified. This limitation is important when interpreting the observed birth prevalence and comparing this data to other sources.

Congenital anomalies identified in the study were categorised into major and minor conditions. Major or serious anomalies are those that may result in death, limited life expectancy or lifelong disability, particularly in the absence of care [9]; whereas minor anomalies may have little impact on health status or quality of life [9,20].

### Case ascertainment and data collection

Relevant cases for inclusion in this study were actively ascertained by the study team. Details of all deliveries at EDH were recorded in the birth register maintained by the Obstetric team. All live-born neonates were examined within 24 hours of delivery before discharge by a midwife or a doctor in the NCU team as a part of routine clinical care. For the study duration, the medical team comprised full-time neonatologists and general paediatricians, with the daily care team also including rotating paediatric registrars, medical officers (3-monthly) and interns (monthly). Following this assessment, healthy neonates and those diagnosed with minor congenital anomalies not requiring further care remained with their mothers in the post-natal units. Neonates and mothers with no complications were discharged as early as six hours post-delivery. Others may have been observed and routinely discharged within 24–48 hours of delivery (longer for weekend deliveries) when they were deemed fit. Sick neonates and those with serious congenital anomalies were admitted to the NCU for holistic management. This included a clinical assessment to ascertain the extent of the abnormality, to undertake appropriate diagnostic testing, clinical care, genetic counselling for parents and notification using the BDNT. These admissions were recorded in the neonatal admission register. All routine activities were managed by the NCU team. For complex cases, the team had access to a general paediatrician with a special interest and some training in clinical genetics for assistance with diagnosis, care and/or genetic counselling. A gatekeeping system in place for appropriate genetic testing ensured referral to and/or consultation with relevant genetic specialists at the tertiary hospital.

Data for this retrospective study were extracted from the birth register, neonatal admission register and the individual BDNT forms (all paper-based). Incomplete BDNT forms or more complex cases requiring a dysmorphology evaluation and syndrome identification (e.g., multiple anomalies) required the researcher to review individual clinical records and update the BDNT forms. All original BDNT forms were routinely kept on file within the NCU. As reported by Lebese et al [15], and during the feasibility assessment for this study it was important to ensure that the BDNT was accurately and fully completed for all identified cases with congenital anomalies to enhance data quality. To achieve this, the researcher/first author clinically rotated through the NCU for three months during the study period and served as a local champion for the span of the project. This was to strengthen active surveillance by raising awareness around congenital anomalies, the BDNT surveillance process, reminding staff to complete the tool and to review the completeness of the forms monthly. Completed BDNT forms were routinely submitted to the hospital coordinator as required by the NDoH. The functionality of this system outside EDH was beyond the scope of the study.

### Data analysis

The in-facility birth prevalence rate was calculated in 2018 using the number of live births with congenital anomalies as the numerator and the total number of live births at EDH as the denominator, multiplied by 1 000 to report the rate per 1 000 live births as per convention. The number, percentage and birth prevalence rates of major, minor (i.e., polydactyly) and isolated congenital anomalies in diagnostic sub-categories were reported separately. To prevent double-counting, neonates with syndromes and multiple anomalies were counted only once in the overall live birth prevalence in the relevant sub-category and not for each specific anomaly. For example, a neonate with Trisomy 21 with a ventricular septal defect and a trachea-oesophageal fistula was counted under Down syndrome only and not separately (again) under both circulatory and digestive systems.

Statistical analysis was undertaken to compare birth prevalence rates for selected congenital anomalies observed with modelled birth prevalence rates for SA published previously [3,21]. Differences between these birth prevalence rates (*p*-values) were calculated using Chi^-^squared statistical test and confidence intervals were calculated with the “test-based method” [22] using the SciStat online calculator (www.scistat.com).

### Ethics

Ethical clearance was granted for this study by the University of KwaZulu-Natal Biomedical Research Ethics Committee (Ref No. BE409/18). Gatekeeper permission to conduct the study was obtained from the Chief Executive Officer at EDH and the study was registered on the National Health Research Database. All data were collected retrospectively in March 2019 from routine care records or registers during the study period and were anonymized at the point of collection with a sequential study number allocated to protect all patients’ identities and personal records. The data collection tool used was an anonymized copy of the BDNT. There was no direct patient contact in this study therefore individual patient consent was not required for ethical clearance. Collected data was stored electronically on password-protected drives and computers.

## Results

Over the 12-month study period, 117 neonates were diagnosed and notified with congenital anomalies from the 7 516 live births examined at EDH. The birth prevalence (of major and minor congenital anomalies) for this period was 15.57 per 1 000 live births. This equates to 1 in every 64 live births affected by a congenital anomaly at EDH in 2018. Excluding polydactyly—a minor congenital anomaly—the birth prevalence decreased to 13.44 per 1 000 live births, equivalent to 1 in 74 births.

The demographic characteristics of the affected births are outlined in Table 1. The demographic characteristics of the total births were not feasible for inclusion in this study due to the large number (7516) and the records being paper-based.

**Table 1:** Demographic characteristics of new-borns affected by congenital anomalies at Edendale Hospital, 2018 (study population).

Category	Characteristic	Number (n = 117)	Proportion (%)
Gender	Male	60	51,3%
	Female	54	46,2%
	Ambiguous	3	2,6%
Population Group	African	116	99,1%
	Other	1	0,9%
Birth Weight	<2500g	51	43,6%
	>2500g	66	56,4%
Gestational Age	<37 weeks	48	41,0%
	>37 weeks	69	59,0%
Advanced Maternal Age	≥35 years	20	17,0%
	<35 years	68	58,0%
	Not recorded	29	25,0%

Congenital anomalies were more prevalent in male, term neonates with normal birth weights (>2500g). Twenty (17%) of the 117 affected births were recorded to mothers of advanced maternal age (AMA) defined as equal to or greater than 35 years of age. However, in 29 affected cases (25%) maternal age was not recorded in the Obstetric births register.

Reported congenital anomalies categorised according to the ICD-10 classification are detailed in Table 2. Anomalies of the musculoskeletal system were most frequently observed, accounting for just under a third (31.6%) of total anomalies recorded in the study period. Excluding polydactyly reduced the proportion of musculoskeletal anomalies to 17.9%, just less than the proportion recorded for the circulatory system (18.8%).

**Table 2:** Birth prevalence and proportion of observed congenital anomalies among live births at Edendale Hospital, SA in 2018.

System/Syndrome	Classification	Aetiology	Number (n = 117)	Percentage (%)	Birth Prev. per 1 000 Live Births
Musculoskeletal			37	31,6%	4,92
	Postminimus Polydactyly^a^	Single Gene Disorder	16		2,13
	Congenital Talipes Equinovarus^b^	Constraint/Multifactorial	11		1,46
	Gastroschisis	Multifactorial	3		0,40
	Achondroplasia	Single Gene Disorder	3		0,40
	Omphalocoele	Multifactorial	2		0,27
	Thanatophoric dysplasia	Single Gene Disorder	1		0,13
	Prune Belly Syndrome	Unknown	1		0,13
Circulatory System			22	18,8%	2,93
	Ventricular Septal Defect	Multifactorial	8		1,06
	Atrial Septal Defect	Multifactorial	5		0,67
	Atrioventricular Septal Defect	Multifactorial	3		0,40
	Patent Ductus Arteriosus	Multifactorial	3		0,40
	Tetralogy of Fallot	Multifactorial	2		0,27
	Pulmonary Stenosis	Multifactorial	1		0,13
Chromosomal			15	12,8%	2,00
	Down Syndrome (T21)^b^	Chromosomal Abnormality	13		1,73
	Edwards Syndrome (T18)	Chromosomal Abnormality	1		0,13
	Patau Syndrome (T13)	Chromosomal Abnormality	1		0,13
Nervous System			9	7,7%	1,20
	Anencephaly^b^	Multifactorial	3		0,40
	Spina Bifida (Meningomyelocoele)^b^	Multifactorial	2		0,27
	Arnold Chiari Malformation—Hydrocephalus	Multifactorial	1		0,13
	Congenital Hydrocephalus^b^	Multifactorial	1		0,13
	Dandy Walker Syndrome	Multifactorial	2		0,27
Digestive System			7	6,0%	0,93
	Tracheo-oesphageal Fistula	Multifactorial	2		0,27
	Duodenal Atresia	Unknown	1		0,13
	Small Bowel Atresia (Jejunal)	Unknown	2		0,27
	Jejunal Atresia—Type 4	Unknown/Multifactorial	1		0,13
	Small Bowel Malrotation	Unknown	1		0,13
Orofacial Clefts (Isolated)			4	3,4%	0,53
	Cleft lip^b^	Multifactorial	2		0,27
	Cleft lip & palate^b^	Multifactorial	2		0,27
Eye, Ear, Face and Neck			3	2,6%	0,40
	Treacher Collins Syndrome	Single Gene Disorder	1		0,13
	Facial dysmorphism^a^	Unknown	2		0,27
Genital System			3	2,6%	0,40
	Ambiguous Genitalia (DSDs)	Multifactorial	2		0,27
	Hypospadias	Multifactorial/unknown	1		0,13
Respiratory System			3	2,6%	0,40
	Choanal Atresia	Unknown	2		0,27
	Congenital Cystic Lung	Unknown	1		0,13
Skin			3	2,6%	0,40
	Neurofibromatosis	Single Gene Disorder	1		0,13
	Epidermolysis bullosa	Single Gene Disorder	1		0,13
	Tuberous Sclerosis	Single Gene Disorder	1		0,13
Other Congenital Disorders & Multiple Malformations			11	9,4%	1,46
	VACTERL Association	Multifactorial	3		0,40
	Foetal Alcohol Syndrome	Teratogen	2		0,27
	Foetal Warfarin Syndrome	Teratogen	1		0,13
	Cornelia De Lange Syndrome	Single Gene Disorders	1		0,13
	Pentalogy of Cantrell	Unknown	1		0,13
	Ambiguous Genitalia & Imperforate Anus	Unknown	1		0,13
	Club feet & facial dysmorphism (Possible Trisomy)	Unknown	1		0,13
	Imperforate Anus + Club feet (Possible VACTERL)	Unknown	1		0,13
**Total**			**117**	**100,0**%	**15,57**

^a^ Minor congenital anomaly.

^b^Designated as a priority CD in SA [13,14].

Polydactyly was the most common individual condition observed, accounting for 13.7% of total congenital anomalies identified. The other frequently reported conditions were Down syndrome (DS, 11.1%) Congenital Talipes Equinovarus (9.4%) and anomalies of the nervous system (7.7%), including isolated Neural Tube Defects (NTDs, 5.1%). Equal numbers of isolated Cleft Palate (CP) were reported as for isolated cleft lip/cleft lip and palate (collectively 3.4%).

Other than chromosomal disorders, the aetiology of congenital anomalies observed in this study were mainly malformations due to multifactorial or unknown reasons. Some were less visible, internal malformations, including congenital heart defects (CHDs). Some single gene disorders with obvious dysmorphic phenotypes were also reported.

Eight (9.4%) of the affected neonates were diagnosed with multiple congenital anomalies. In some cases, these were recognisable, dysmorphic syndromes caused by teratogen exposure during pregnancy (foetal warfarin syndrome and foetal alcohol syndrome), known genetic mutations (Cornelia De Lange syndrome) or due to associated congenital malformations including Vertebral-Anorectal-Cardiac-Tracheo-Esophageal-Renal-Limb (VACTERL) association and Pentalogy of Cantrell, due to unknown causes.

The proportion of affected neonates that underwent investigations are summarised in Table 3. Of the 117 affected neonates identified, 96 (82%) underwent relevant blood and imaging investigations including hormonal testing, congenital infection screening, x-rays, ultrasound, echocardiography or computed tomography (CT) scans; 25 underwent Trisomy Polymerase Chain Reaction (PCR) blood testing, of which 15 (60%) were confirmed as Trisomy 13, 18 or 21. A further nine neonates had karyotype testing with seven normal results and in two cases the results were lost by the testing laboratory.

**Table 3:** Summary of investigations undertaken on neonates with congenital anomalies.

Investigation	Number (n = 117)	Proportion (%)	Investigation
Relevant investigation	96	82%	Blood & Radiology
Chromosomal Analysis (Trisomy PCR)	25	21%	15 (60%) confirmed trisomy
Biochemical Analysis	0	0%	Not recorded on the form
DNA/Molecular Analysis	0	0%	N/A in KZN at the time of the study
Karyotype	9	8%	7 were normal, 2 lost by laboratory

## Discussion

This study measured the birth prevalence of congenital anomalies among live births at EDH in KZN, SA from January to December 2018. Examination of all new-borns and recording of anomalies identified at birth (by discharge) were reported using the enhanced BDNT for notification to the NDoH. Data collected were described and compared with existing published data for congenital anomalies, including historic research studies and modelled estimates for SA [3].

### Birth prevalence

Birth prevalence studies for congenital anomalies may be useful to establish baseline rates and monitor trends, explore possible causes, develop prevention strategies, and review changes and response to interventions for specific anomalies over time.

The main aim of this study was achieved with the measurement of the birth prevalence of congenital anomalies that were easily recognisable by discharge after birth as a subset of total CDs possible during all life stages. When compared with previously published birth prevalence rates for congenital anomalies globally; averaging at 20–30 per 1 000 live births [23–26], both the rate observed (15.57 per 1 000 live births) and the rate excluding polydactyly (13.44 per 1 000 live births) in this study are approximately half the published average birth prevalence of *congenital anomalies*. This may be due to differences in the scope and case definitions used in these studies. Poorer case identification at the study site for reasons highlighted as study challenges and limitations may also be a factor.

The observed birth prevalence rate in this study is very similar to another SA study by Venter et al (14.97 per 1 000 live births in 1995), published over 25 years earlier [26]. This offers the opportunity for potential comparison of congenital anomalies data changes in SA before HIV/AIDS and after its successful prevention (PMTCT) in neonates. It is also comparable to previously published data from two other LMIC sites in Africa at different times using similar methodologies and case definitions, specifically in Uganda by Tann et al (17 per 1 000 live births in 2006) [27] and Abbey et al in Nigeria (20.73 per 1 000 live births in 2017) [28].

If a congenital anomalies birth prevalence of 15.57 per 1 000 live births represents the 26% that are easily recognisable at birth [26], then in a relatively constant catchment population at EDH the *total* CD-related birth prevalence for the study site may be approximated to around *59*.*88 per 1 000 live births*. This is again within the range quoted for *total* CD-related birth prevalence globally of between 40 to 80 per 1 000 live births [9]. The modelled value for SA using 2012 as the reference year published by Malherbe et al was specified as 68 per 1 000 live births [21]. This suggests the observed birth prevalence of congenital anomalies at EDH is likely to be close to the true value. An extension of this study measuring the birth prevalence over the different age periods (neonatal, infant and under-five) would offer a clearer picture of the total CD burden.

### The pattern of congenital anomalies

#### Previous published SA studies

**Fig 1:**
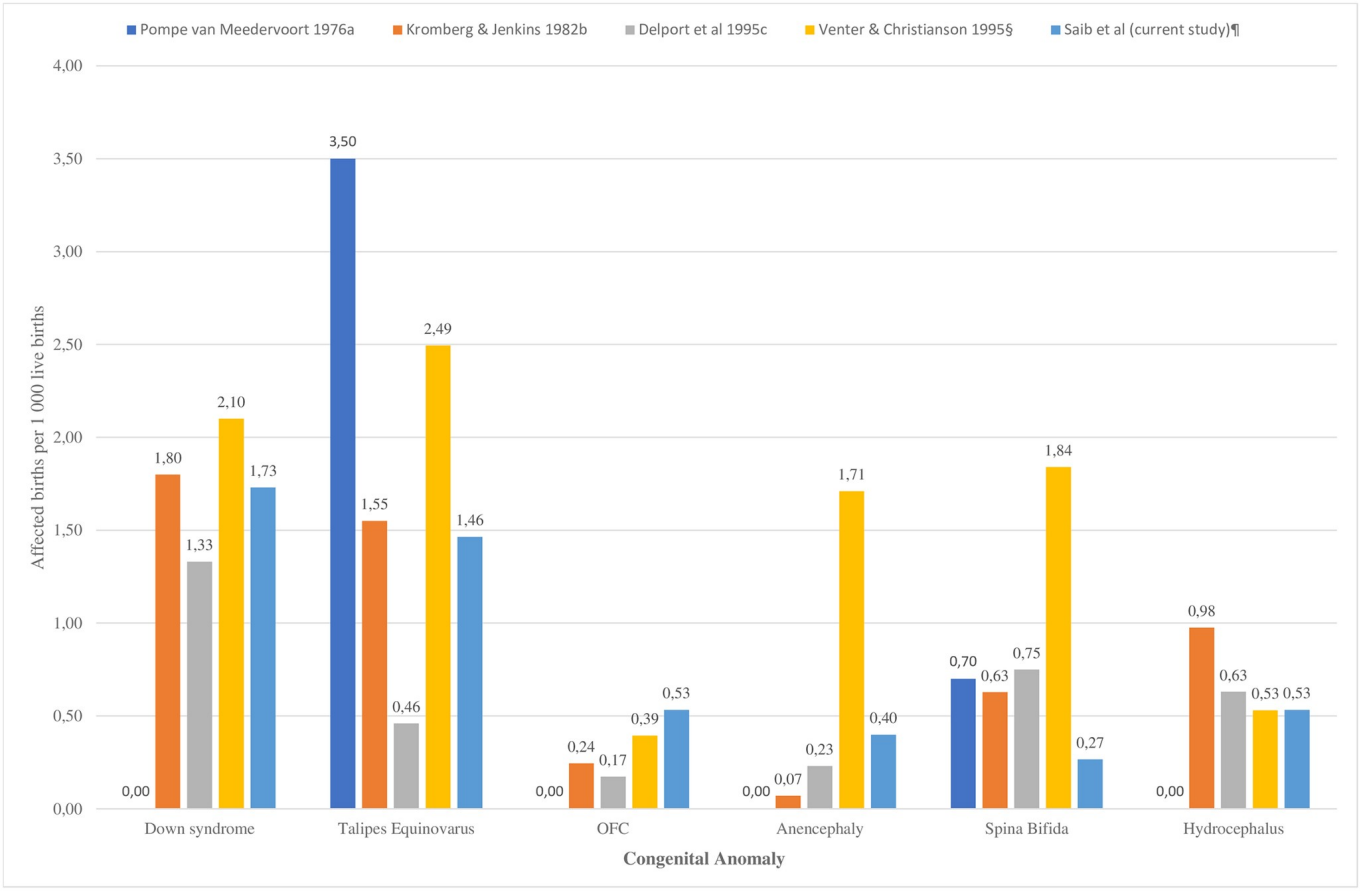
Comparison of birth prevalence rates for key congenital anomalies from the current study with rates observed by other facility-based studies in South Africa. ^a^ Pompe van Meedervoort 1976: Prospective,75% urban hospital-based study (Pelonomi Hospital, Bloemfontein, Free State), 10 000 live births over 3 years [23]. ^b^ Kromberg & Jenkins 1982: Retrospective, urban hospital-based study (Chris Hani Baragwanath Hospital, Johannesburg, Gauteng), 28 689 live births over 2 years (1976 to 1977) [24]. ^c^ Delport et al 1995: Prospective, urban hospital-based study (Kalafong Hospital, Pretoria, Gauteng), 17 351 live births over 3 years (1986 to 1989) [25]. ^d^ Venter & Christianson 1995: Prospective, rural, hospital-based (Mankweng Hospital, Limpopo), 7 617 live births over 3.5 years (1989 to 1992) [26]. ^e^ Saib et al 2021: Retrospective, predominantly urban, hospital-based (Edendale Hospital, Pietermaritzburg, KwaZulu Natal), 7 516 live births over one year (2018, current study).

The pattern of congenital anomalies observed in this study aligned with those reported globally. The most frequently reported conditions are all defined as priority conditions in SA by the NDoH [13,14]. Adding further credibility was the comparison of birth prevalence rates of key congenital anomalies with observed with rates obtained through similar facility-based South African studies, presented graphically in Fig 1. The highest birth prevalence rate was observed by Venter and Christianson et al in the only rural study for three of the congenital anomalies profiled (DS, anencephaly and spina bifida) [26]. The highest overall birth prevalence was recorded for Congenital Talipes Equinovarus by Pompe van Meerdervoort et al in the 1970s [23]. While the birth prevalence rates were comparable for most anomalies, it was lowest for spina bifida and highest for Orofacial Clefts (OFC).

The high variation in birth prevalence of NTDs between the studies may be attributed to the introduction of mandatory folate fortification of staple crops in 2003 [29,30]. Similarly, higher NTD rates reported by Kromberg and Jenkins in 1982 may be indicative of improved reporting via a retrospective approach, compared to the other prospective studies [24]. This alludes to paradoxically poorer data collection in prospective versus retrospective methods due to the study challenges highlighted later in this article. Reasons for the high rates of spina bifida and anencephaly reported by Venter and Christianson et al in comparison to other studies pre-dating folate fortification are still not clear and may be attributed to the complex interaction of genetic and environmental factors [26,31,32]. The extremely high birth prevalence reported by Pompe van Meedervoort et al for Congenital Talipes Equinovarus may be due to the inclusion of both isolated and syndromic club foot whereas the current study differentiates between these aetiologies (case definitions) [23].

Based on previously reported birth prevalence rates by similar studies for oculocutaneous albinism; one of the commonest single-gene disorders in SA, at least 2 affected births would have been expected in this study [24–26]. This absence cannot be explained. Differences in birth prevalence rates of DS and other Trisomies may reflect variations in the proportions of mothers of AMA in the studies, as well as challenges in identifying neonates with these conditions at birth [33,34]. Due to incomplete data on AMA in the current and previous studies, further analysis was not possible.

### Modelled SA data

In Table 4, the live birth prevalence rates observed in the current study were compared to modelled national estimates for SA in 2017 generated via the Modell Global Database of Congenital Disorders (MGDb) [3,6,35–37]. The MGDb method combines robust, observed data from well-established surveillance systems with demographic data to produce baseline (no interventions) and actual (current care) estimates, using the infant mortality rate (IMR) as a proxy to quantify available services [3,6,35].

**Table 4:** Comparison of observed (EDH) with modelled (SA) birth prevalence rates of selected congenital anomalies.

Condition^a^	Saib et al (current study)		Modell et al 2016 [3]		p-Value^c^
	Birth Prevalence Rate (per 1000 live births)	Confidence Interval (95%)	Birth Prevalence Rate (per 1000 live births	Confidence Interval^b^ (95%)	
**Down syndrome**	1.73	0.92–2.96	1.60	1.53–1.68	0.78 (ns)
**Neural Tube Defects**^d^	0.67	0.29–1.74	0.85	0.79–0.90	0.89 (ns)
**Orofacial Clefts**	0.53	0.15–1.36	0.22	0.19–0.25	0.07 (ns)
**Congenital Heart Defects**	2.93	1.83–4.43	3.15	3.05–3.25	0.73 (ns)

^a^ Isolated congenital anomalies only are included in this data comparison.

^b^ For the confidence intervals (CI) of the difference between the two birth prevalence rates, the test-based method was used (SciStat.com) [22].

^c^ *p*-values were calculated using the Chi^2^-statistic (SciStat.com) [22].

^d^ Neural Tube Defects (NTDs) were limited to isolated anencephaly, spina bifida and hydrocephalus. For the current study other syndromes associated with hydrocephalus, e. g. Dandy-Walker syndrome and Arnold Chiari malformation observed in this study (Table 2) were excluded as these are not isolated congenital anomalies.

While there was apparent variation between birth prevalence rates for the conditions compared via the observed and modelled data (Table 4), statistical analysis revealed no significant difference between any of the compared rates. This comparison has limitations due to differences in demographics, e. g. year of study (2017 versus 2018) and the population (SA versus KZN facility) and specific indicators vary locally versus nationally. Nevertheless, this comparison may serve to validate the MGDb modelling approach, including results via the SA application [21] for the key congenital anomalies at sites that lack the ability or resources to conduct routine surveillance or research.

### Study strengths

This study, one of only a few undertaken following the improved management of the HIV/AIDS crisis, confirms congenital anomalies as a significant healthcare burden for SA to address. This was the first study conducted in KZN to determine the birth prevalence of congenital anomalies, with all previous published studies undertaken in other provinces. This empiric evidence highlights the re-emergence of congenital anomalies (and CDs) in KZN and the need for similar studies in other parts of the country.

This facility-based study, incorporating active surveillance, is more feasible for large, diverse populations, particularly in LMICs such as SA where healthcare resources (both human and financial) are constrained. While this type of study may be subject to referral bias due to births by non-residents and referrals from outlying clinics before birth, it offers the advantage of obtaining high-quality data on key conditions at sentinel sites. The alternative, population-based approach is unaffected by this type of bias as it includes all (home and facility-based) deliveries. However, these studies are more suited for HIC, and smaller populations or regions or by sub-national systems due to the higher cost and infrastructure required, as has been demonstrated by poor data resulting from the population-based BDNT implemented in SA [15].

The collection of accurate data is essential for precise surveillance. Before the study, a passive surveillance approach yielded approximately 3–6 congenital anomalies per 600 deliveries on average per month at the study facility (Personal communication Dr Bhoola, Head of Clinical Unit—NCU 2017). In this study, an active surveillance approach supported by a clinical champion resulted in a higher rate of congenital anomalies being detected, averaging at 10 per 600 deliveries per month. This simple and effective quality improvement measure enhanced reporting accuracy and offers a feasible methodology for sites in other LMIC contexts. Good quality data is more useful when comparing with other studies.

### Study challenges

Before a congenital anomaly can be reported it must first be accurately and timeously diagnosed. This requires appropriately trained healthcare professionals (HCPs) able to diagnose congenital anomalies and comply with reporting requirements of a surveillance system as a part of routine clinical care. Genetics content included in both medical school and nursing college curricula is insufficient [38–40], and varies greatly between institutions and countries, resulting in many HCPs lacking relevant genetics knowledge, skills, and expertise.

In SA, specialised medical genetic services are inadequate. There were only 12 practising clinical medical geneticists and a similar number of genetic counsellors countrywide in 2015, equating to 1 per 5 million and 1 per 8.4 million of the population respectively [41]. This is five times below the recommended number per population of 1 per million for clinical medical geneticists and 1 per 580 000 for genetic counsellors [41,42]. There have been minimal changes in these capacity ratios in recent years due to retirement, emigration and lack of state-posts for qualifying HCP in this sector. Despite being without a medical geneticist for decades, and the loss of an effective provincial coordinator in 2012, KZN was reported as the province with the highest reporting compliance, contributing over 50% of national surveillance data [15]. In 2018, following intense advocacy efforts, one medical geneticist was hired for the 11 million population of the KZN province and a centralised provincial genetic service is now being developed out of the tertiary hospital in Durban. Recruitment of a genetic counsellor is underway with ongoing support of two genetic nurses.

In this study (prior to the appointment of a medical geneticist in province), complex cases were referred to a paediatrician with a genetics interest in place of a medical geneticist. This individual has received training in the Medical Genetics Education Program (MGEP) and five years of experience running a specialist level referral clinical genetics clinic at the nearby tertiary hospital in Pietermaritzburg. The team of neonatologists have received no formal genetics training other than their sub-speciality training, which included access to a foeto-maternal anomaly clinic at the training site. The remainder of the medical and nursing teams lack specialised genetics training. While the gatekeeping system involving the referral of patients to genetic specialists at tertiary hospitals in the region ensures appropriate tests are requested, it places additional stress on an already poorly resourced system.

The use of paper-based systems, including the BDNT, continues to impact surveillance compliance and data quality even when integrated as part of routine clinical care. Within this study, the active, champion-based surveillance effectively improved data quality. However, a sustainable, long-term solution is needed, through the investment of relevant resources into integrating the notification of congenital anomalies as part of an effective health information system and electronic health record. By default, this would fill data gaps experienced in this study, such as missing maternal age data to establish the true extent of AMA as a risk factor for Trisomies. Such data systems are already in place in most private facilities and billing through medical aid (insurance) schemes (including ICD-10 coded diagnosis) making this smaller sector (approx. 15% of health services in SA) appealing as a feasible and additional source of reliable CD data, despite not currently contributing to the BDNT.

Some blood samples of the study population were lost by the testing laboratory. Anecdotal evidence suggests this is a common challenge in the province and largely systemic, with loss and leakage of samples occurring during transport from satellite to main laboratory sites. The additional cost implications, delayed diagnosis and patient trauma due to repeated testing highlight the need for these challenges to be addressed.

These challenges may have contributed to the lower birth prevalence observed and highlight the urgent need for capacity building at all levels, together with appropriate resource investment to improve the surveillance, care and prevention for those affected by all CDs.

## Limitations

This study was limited to recording live births affected by congenital anomalies and excluded affected births (live births and stillbirths) occurring outside EDH, from both public and private facilities. The proportion of ‘missed’ congenital anomalies resulting in stillbirths and early pregnancy losses, including termination of pregnancy, remains an unquantified element of the CD burden in this setting.

The focus of the study on congenital anomalies only, a sub-set of CDs, excluded a significant proportion included elsewhere in the ICD-10 system such as inborn errors of metabolism and other single gene disorders. This prevented the measurement of the *total* CD-related burden [3,18]. A further example, Foetal Alcohol Spectrum Disorder (FASD) is challenging to diagnose at birth, with most cases being picked up in school-age children, limiting early diagnosis to the most severe cases presenting with an obvious phenotype. Congenital infection data and other risk factor data, including in-utero exposure to antiretroviral drugs was also not collected.

## Conclusion

This study on congenital anomalies at birth is the first published from KZN and the most recent in SA after the successful implementation of the PMTCT program. It quantified the birth prevalence of live births affected by congenital anomalies at EDH in 2018, demonstrating the ability of this facility-based method to collect high quality, accurate data on these conditions. The study responded to the paucity of available birth prevalence data on congenital anomalies in SA and has demonstrated the impact of championed, quality improvement efforts addressing diagnosis and notification challenges. It also offers reassurance that this approach may be replicated in similar contexts. The observed study rates are in line with published data locally and globally and serve as a baseline for the comparison of trends over time. The rates are also consistent with modelled estimates, indicating the further application of MGDb and other modelling approaches in under-served areas that lack resources to measure accurate data.

This study offers additional evidence on the health burden represented by congenital anomalies in SA and the need to prioritise these conditions, their surveillance, care and prevention, as a healthcare priority. To respond appropriately to the proportionately growing health burden of congenital anomalies as infectious diseases are better controlled in SA, further studies of this nature are required to offer policy-makers reliable evidence for informed data-based decision making around essential healthcare services and value-based allocation of available limited funding. This should be undertaken in tandem with investment in electronic surveillance systems if SA is to respond appropriately to specific local, regional, and national health needs to prevent people with CDs from being excluded.

Further research is recommended on:

1. Increased scope of study to include:
  - Congenital anomalies among stillbirth, pregnancy losses and termination of pregnancy at the study site.
  - Affected births from referring facilities within the catchment area to enable a birth prevalence rate closer to the true value.
  - Follow up population prevalence studies to incorporate other life-course stages including neonates, infants and children affected by congenital anomalies and other CDs beyond the birth period to improve quantification of the total CD burden of disease.
  - Commonly missed functional CDs in the ICD-10 system in addition to structural disorders.
  - Similar studies in other regions of SA to enable comparison to identify regional and demographic similarities or differences.
  - The private healthcare setting using existing information systems to complete the picture for the entire SA population or looking at access and outcomes in the different sectors (insured vs uninsured).
  - Looking at clinical care and outcomes of patients affected by CDs treated at hospitals, and how to improve quality through interventions such as early antenatal identification and planning of services before birth.
2. Capacity building for HCPs on the clinical and genetic diagnosis of congenital anomalies and other CDs and reporting strategies, including the BDNT, to promote improved diagnosis and more accurate, comprehensive reporting.
3. Long-term options for improving congenital anomaly and wider CD reporting i. e., the inclusion of congenital anomalies on the neonatal dashboard as a sentinel group of disorders and electronic health records as part of an integrated health information system, to evaluate the return on investment enabled through better diagnosis and care.

## Supporting information

S1 File Data set. (XLSX)
